# Three-Dimensional Visualization With Tissue Clearing Uncovers Dynamic Alterations of Renal Resident Mononuclear Phagocytes After Acute Kidney Injury

**DOI:** 10.3389/fimmu.2022.844919

**Published:** 2022-03-10

**Authors:** Kipyo Kim, Yun-Gyeong Kim, Su Woong Jung, Yang Gyun Kim, Sang-Ho Lee, Seung-Hae Kwon, Ju-Young Moon

**Affiliations:** ^1^Division of Nephrology and Hypertension, Department of Internal Medicine, Inha University School of Medicine, Incheon, South Korea; ^2^Division of Nephrology, Department of Internal Medicine, Kyung Hee University, College of Medicine, Seoul, South Korea; ^3^Korea Basic Science Institute, Seoul Center, Seoul, South Korea

**Keywords:** acute kidney injury, innate immunity, mononuclear phagocyte, tissue clearing methods, light sheet fluorescence microscopy

## Abstract

Traditional histologic methods are limited in detecting dynamic changes in immune cells during acute kidney injury (AKI). Recently, optical tissue clearing combined with multiphoton microscopy (MPM) or light sheet fluorescence microscopy (LSFM) has become an emerging method for deep tissue evaluation and three-dimensional visualization. These new approaches have helped expand our understanding of tissue injury and repair processes, including tracing the changes in immune cells. We designed this study to investigate the morphological and functional alterations of renal mononuclear phagocytes (MNPs) in lipopolysaccharide (LPS)-induced AKI using renal clearing in CD11c-YFP mice. We also evaluated the effect of the NLRP3 inhibitor MCC950 to determine whether NLRP3 inhibition attenuates the activation of CD11c+ cells in an LPS-induced AKI model. Transverse sectioned whole mouse kidney imaging by LSFM showed that CD11c+ cells were mainly distributed in the cortex, especially the tubulointerstitial area. The number of CD11c+ cells was significantly more densely interspersed, particularly in periglomerular and perivascular lesions, in the saline-treated LPS-exposed kidney than in the control kidney. Deep imaging of the kidney cortex by MPM demonstrated an increased number of CD11c+ cells in the saline-treated LPS group compared with the control group. This quantitative alteration of CD11c+ cells in AKI was accompanied by morphological changes at high resolution, showing an increased number and level of dendrites. These morphological and behavioral changes in the saline-treated LPS group were accompanied by increased MHC class II and CD86 on CD11c-YFP+ cells. MCC950 attenuated the activation of CD11c+ cells after AKI and improved renal function. In conclusion, wide and deep three-dimensional visualization using MPM or LSFM combined with kidney clearing uncovers dynamic changes of renal MNPs, which are directly linked to renal function in AKI.

## Introduction

Acute kidney injury (AKI) is a complex syndrome implicated in various clinical conditions and etiologies. A growing body of evidence indicates a central role of inflammation in the pathophysiology of AKI (1). Almost all immune cells in both the innate and adaptive immune systems are involved in AKI processes (2). Of these, renal resident mononuclear phagocytes (MNPs), such as monocytes, macrophages, and dendritic cells (DCs), have pivotal roles in maintaining immunologic homeostasis in the steady state and are implicated in the relatively early phase of AKI (3). Therefore, renal MNPs are an important research topic in alleviating AKI and chronic kidney disease (CKD) transition.

The number, location, morphology, and characteristic functional changes of renal MNPs from steady-state conditions to AKI have been evaluated mainly with histological staining and flow cytometry. Despite the technical advances in histological staining methods, quantitative and morphological analyses, especially those regarding the three-dimensional (3D) structure of resident and recruiting renal MNPs, are challenging. Furthermore, the processes of histological analysis, such as embedding, sectioning, staining, and manual counting, are laborious and time-consuming. This approach also has difficulty providing precise morphological information and results in loss of spatial data of renal MNPs. The development of clearing techniques and analysis of cleared organs with various microscopies, such as light sheet fluorescence microscopy (LSFM) or multiphoton microscopy (MPM), has enabled us to overcome the current unmet needs of histological analytic methods. These techniques have led to brain research on the 3D shape and the route of neurons and blood vessels in the whole brain from the cortex to the pituitary gland (4). This strategy is a revolutionary technique that will improve the tracking and reconstruction of neuronal axons and their branches (5). The kidney is also an anatomically complicated organ for evaluating the morphological and functional changes of each cell type after injury, even inside the cortex.

Since MNPs express the essential components of the NLRP3 inflammasome, they are critical players in Nod-like receptor family, pyrin domain-containing protein 3 (NLRP3) inflammasome activation (6). There is growing evidence that NLRP3 activation is closely associated with the innate immune response in AKI of various etiologies (7). In animal models of sepsis-induced AKI, the expression levels of NLRP3, apoptosis-associated speck-like protein containing a CARD, and caspase-1 were upregulated, and renal injury was ameliorated by NLRP3 deficiency or caspase-1 inhibition (8, 9). Several NLRP3-targeted agents have been investigated in *in vitro* studies, some of which effectively inhibit the NLRP3 inflammasome pathway in MNPs (10). MCC950 inhibits canonical and noncanonical NLRP3 inflammasome activation by blocking the ATPase domain of NLRP3 (11), exerting protective effects in models of AKI, such as ischemia-reperfusion injury (IRI) and sepsis-induced AKI (12–14).

We performed this study to evaluate the quantitative and qualitive alterations of renal MNPs in a murine model of lipopolysaccharide (LPS)-induced AKI with CD11c-yellow fluorescent protein (YFP) mice using MFM and LSFM analysis after kidney clearing. We also assessed the effect of the NLRP3 inhibitor MCC950 on renal MNPs with these approaches.

## Materials and Methods

### Murine Model of Lipopolysaccharide-Induced AKI

CD11c-YFP (Itgax-Venus) mice and C57BL/6 mice were purchased form The Jackson Laboratory (USA) and Junbiotech (Gyeonggi, Korea). CD11c-YFP mice were used for imaging experiments, while C57BL/6 mice were used for histological analysis and immunofluorescence staining for F4/80. To induce acute kidney injury, CD11c-YFP mice were intraperitoneally injected with LPS (5 µg/g), whereas the control group (n=7) was injected with the same volume of 0.9% saline. The LPS-treated mice were pretreated with either MCC950 (10µg/g, intraperitoneally, n=9) or 0.9% saline (n=7) 1 hour before LPS administration. Mice were euthanized, and kidneys were harvested 24 hours after LPS treatment. Mice received 10 μg of Alexa Fluor ^®^ 647 anti-mouse CD31 antibody (BioLegend^®^, 102516, 0.5 μg/μl) *via* the tail vein 10 min before euthanasia. Blood urea nitrogen and serum creatinine levels were determined using the VetTest 8008 (IDEXX Laboratories, Westbrook, ME, USA). All animal experiments were performed in compliance with the guidelines of the Animal Research Ethics Committee of Kyung Hee University Hospital at Gangdong (KHNMC AP 2019-014).

### Histology

Kidney tissues were fixed with 10% neutral buffered formalin at 4°C for 24 hours and embedded in paraffin. Paraffin blocks were cut into 4-μm-thick sections and stained periodic-acid sciff (PAS) reagent. After staining with PAS, morphologic changes were assessed. Tubular epithelial cell vacuolar deformation/hypertrophy and luminal occlusion occurred during renal tubular injury. We assessed tissue injury using the semiquantitative tubular injury score used in previous studies (15, 16). The areas of the tubular epithelial cell vacuolar deformation, loss of brush border, tubular dilation, cast formation and cell lysis were calculated. A score of 0 indicated no change; scores of 1, 2, 3 or 4 indicated an injury section involving <25, 25–50, 50–75, or >75% of the area, respectively. A minimum of ten different fields of vision were randomly selected using a light microscope (magnification x400) and an average score was calculated. For immunofluorescence staining, cryosections were air-dried for 15 min, then primary antibodies against the following proteins were used: F4/80 (rat, 1:50, BioRad, USA). The slides were then exposed to AlexaFluor 488-conjugated goat anti-Rat IgG (1:200) secondary antibodies (Thermo Fisher Scientific, USA). Slides were counterstained with DAPI to delineate the nuclei. Representative images were taken with confocal microscopes LSM-700 (Carl Zeiss, Germany). The number of F4/80+ cells in the tubulointerstitium was counted in ten consecutive fields under high-power fields (x63 oil lens).

### Real Time PCR

RNA was extracted from the kidney tissue using the Total RNA Isolation Kit (Macherey-Nagel, Düren, Germany) and was reverse-transcribed into complementary DNA (cDNA) using random primers (Promega, Madison, WI, USA), deoxynucleotide mix (Takara Bio Inc., Shiga, Japan), and Maloney-murine leukemia virus reverse transcriptase (Mbiotech Inc., Gyeonggi, Korea). Thereafter, real-time PCR was run on the Applied Biosystems^®^ StepOnePlus™ System with a final volume of 20 µl containing 1 µl of cDNA, 5–10 pmol of each of the sense and antisense primers, 10 µl of Power SYBR Green Master Mix (Applied Biosystems, Foster City, CA, USA), and nuclease-free water. The primer sequences used 18S: 5’-GTAACCCGTTGAACCCCATT -3’, 5’ -CCATCCAATCGGTAGTAGCG -3’, IL-6: 5’-ATGGATGCTACCAAACTGGAT -3’, 5’-TGAAGGACTCTGGCTTTGTCT-3’, TGF-beta: 5’-CAACAATTCCTGGCGTTACCTTGG-3’,5’GAAAGCCCTGTATTCCGTCTCCTT-3’, CTGF: 5’ -GAGTGTGCACTGCCAAAGAT -3’ 5’GGCAAGTGCATTGGTATTTG -3’. Each sample was run in duplicate, and data were analyzed using the ∆∆CT method with normalization to 18S expression.

### Kidney Tissue Clearing

We cleared the kidneys with the Tissue Clearing Kit from Binaree (Daegu, Korea). Briefly, formalin-fixed CD11c-YFP mouse kidneys were sectioned into 2-mm-thick tissues. The sections were transferred into the Fixing solution at 4°C for 6 hours and then into the Tissue clearing solution A at 42°C. After 4 days, kidneys were washed with 3mL of Washing solution for 8 hours at 42°C. Next, tissues were transferred in 2mL of Clearing solution B for 2 days at 42°C. Likewise, kidneys were washed with 3mL of Washing solution for 8 hours at 35°C, immersed in 3mL of Mounting solution at 35°C, and maintained for more than 12 hours. After washing, tissue clearing process was continued in the solution B at 42°C for another 2 days. The sections were stored in the Mounting Solution until imaged.

### Imaging and Analysis

We used the light sheet fluorescence microscope (Lightsheet Z.1, Carl zeiss, Seoul Center, Korea Basic Science Institute) equipped with an EC Plan-Neofluar 5x/0.16 objective and clarity chamber (5x, n:1.45). The laser wavelengths used were 488 nm and 638 nm, and the laser intensity was measured under the same conditions. Whole kidney images were constructed from 6~9 tiles with 10% overlap and merged into a single 3D image stack using Arivis software (Arivis AG, Munich, Germany). Three-dimensional rendering of LSFM and laser scanning microscopy data was performed by Imaris 9.5.0 (Bitplane, Switzerland). Quantitative analysis of LSFM data was performed using 3D stacks of 161 images with a size of 863 × 863 × 650 μm acquired from five fields of view in each group. Selected 3D stacks included 40 to 50 glomeruli.

MPM images were acquired using a multiphoton microscope (LSM780 NLO, Zeiss, Germany) with a dual band femtosecond pulse laser system (Spectra Physics InSight DS+, USA) at Seoul Center in Korea Basic Science Institute. For 3D reconstruction of MPM images, ImageJ (National Institutes of Health, Bethesda, MD, USA) and Imaris were used. MPM images were quantitatively analyzed using five 3D stacks of 75 images with a size of 354 × 354 × 150 μm for each group. Single plane data was analyzed with five representative images selected from MPM image stacks. Maximum intensity projection (MIP) images were generated from the stacks of z-plane images using the Z project function of ImageJ. The area of CD11c+ cells was calculated as (CD11c-YFP+ area/total area) × 100. In addition, 3D stacks of 49 MPM images (100 × 100 × 100 μm) acquired from five fields in each group were analyzed for morphometric analysis of dendrites.

Three-dimensional quantitative analysis was performed using Imaris modules. The CD11c-YFP signals were surface-rendered using Imaris surface module. Background subtraction (local contrast) was used, and the surface detail was set to 0.8 μm (smoothness). The upper threshold was determined automatically, and the lower threshold was manually selected to be 10% of the upper threshold. The surface objects for glomeruli were selectively reconstructed from CD31 signals using a diameter of the largest sphere of 65 μm (background subtraction; surface detail, 1.5 μm). Surface objects incorrectly selected as glomeruli were removed using the surface editing tools. The glomerular volume was measured by dividing the total volume of glomerular surface objects by the total number of glomeruli. After surface rendering, unspecific background signals were removed by setting voxels outside surface to zero.

The total number of dendrites and dendrite segments was quantified within the selected 3D stacks. A single cell is defined by its corresponding surface object. Dendrites were selected using autopath algorithm (no loop) of Imaris filament module based on the local contrast; dendrites are defined as sequences of vertices and edges with branching, and dendrite segment indicates the part between branch points (or end points). The starting point (largest diameter) and seed point (thinnest diameter) of dendrites were set to 6 μm and 3 μm, respectively. The thresholds for the starting points and seed points were automatically determined. The threshold for the starting points was adjusted if there were multiple or no starting points in each cell. The total number of dendrites was estimated from the number of dendrite terminal parts provided from the Imaris filament module. Dendrite branch level was hierarchically assigned according to the diameter calculations of individual dendrite segments and averaged by the number of dendrite segments. All parameters of surface and filament objects were exported from Imaris for statistical analysis.

### FACS

Isolated MNPs from kidneys were incubated with surface markers anti-mouse CD86 (PE/Cy7; BD Biosciences) and MHC class II (PE/Dazzle 594; BD Biosciences). Cells were analyzed on a fluorescence-activated cell sorting (FACS) Caliber flow cytometry system (BD Biosciences), and flow cytometric data were analyzed using FlowJo software version 10 (TreeStar, Ashland, Oregon).

### Statistical Analysis

All values are expressed as the mean ± standard error of the mean (SEM) The results were analyzed using the Kruskal-Wallis nonparametric test for multiple comparisons. Significant differences in the Kruskal-Wallis test were confirmed by the Wilcoxon rank sum and Mann-Whitney tests (used to compare mean differences). P values < 0.05 were considered statistically significant.

## Results

### NLRP3 Inhibition Attenuated LPS-Induced AKI

**Figure 1:**
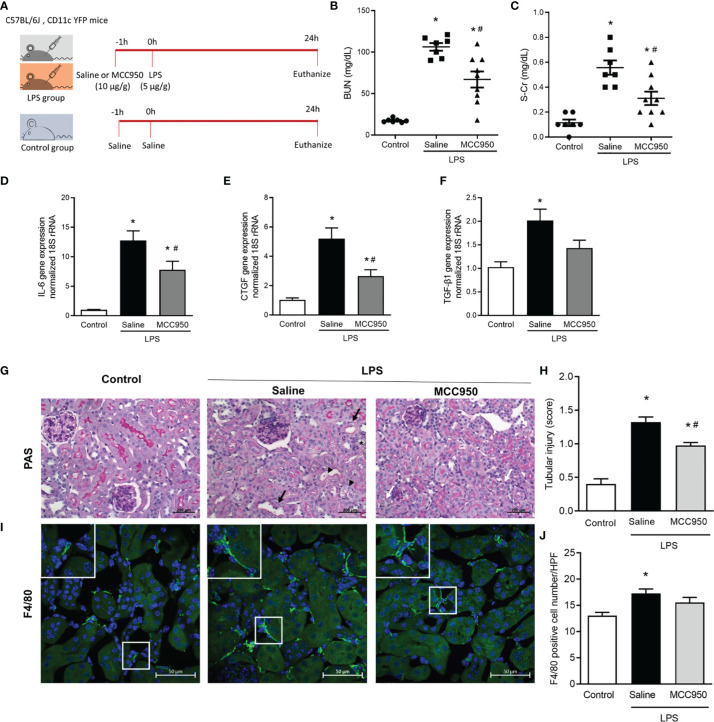
NLRP3 inhibition attenuates LPS-induced AKI and inflammatory cytokine gene expression. **(A)** Experimental design of the study. **(B)** BUN. **(C)** Serum creatinine. Gene expression levels of **(D)** IL-6, **(E)** CTCF, and **(F)** TGF-β1. **(G)** PAS staining shows tubular dilation (black arrows), tubular epithelial cell destruction (black arrow heads), loss of brush border (asterisk), and vacuolar deformation (white arrows). **(H)** Tubular injury score. **(I)** F4/80 staining. **(J)** F4/80+ cell counts. Data are represented as the mean ± SEM; n=7 for control and saline-treated LPS group, n=9 for MCC950-treated LPS group. BUN, blood urea nitrogen; IL-6, interleukin-6; CTCF, connective tissue growth factor; TGF-β1, transforming growth factor-β1; PAS, periodic acid-Schiff; HPF, high power field. *P<0.05 *versus* the normal control, ^#^*P*<0.05 *versus* the saline-treated LPS group.

We used three animal groups for this study: the control, saline-treated LPS, and MCC950-treated LPS groups (**Figure 1A**). The saline-treated LPS group showed higher levels of blood urea nitrogen (BUN) (17.4 ± 2.3 vs. 106.3 ± 12.3 mg/dL; p < 0.05, **Figure 1B**) and serum creatinine than the control group (0.11 ± 0.07 vs. 0.56 ± 0.15 mg/dL; p < 0.05, **Figure 1C**). MCC950 treatment significantly reduced the BUN and creatinine levels compared with those of the saline-treated LPS group (106.3 ± 12.3 vs. 67.0 ± 29.1 mg/dL; p < 0.05, 0.56 ± 0.15 vs. 0.31 ± 0.16 mg/dL; p < 0.05). We also analyzed the tissue expression of interleukin-6 (IL-6), connective tissue growth factor (CTGF), and transforming growth factor-β1 (TGF-β1) to determine the inflammatory and profibrotic processes caused by LPS treatment. The expression of IL-6, CTGF, and TGF-β1 was significantly higher in the saline-treated LPS group than in the control group (**Figures 1D–F**). MCC950 treatment decreased the mRNA expression levels of IL-6 and CTGF in the kidney. The inhibition of NLRP3 by MCC950 ameliorated LPS-induced renal injury and inflammatory cytokine gene expression and resulted in a lower tubular injury score in the MCC950-treated LPS group than in the saline-treated LPS group (**Figures 1G, H**). Immunostaining for F4/80, a marker of monocytes, showed an increased number in the saline-treated LPS group compared to the control group, while there was no difference between the saline-treated and MCC950-treated LPS groups (**Figures 1I, J**).

### Whole Kidney Imaging by LSFM Analysis

**Figure 2:**
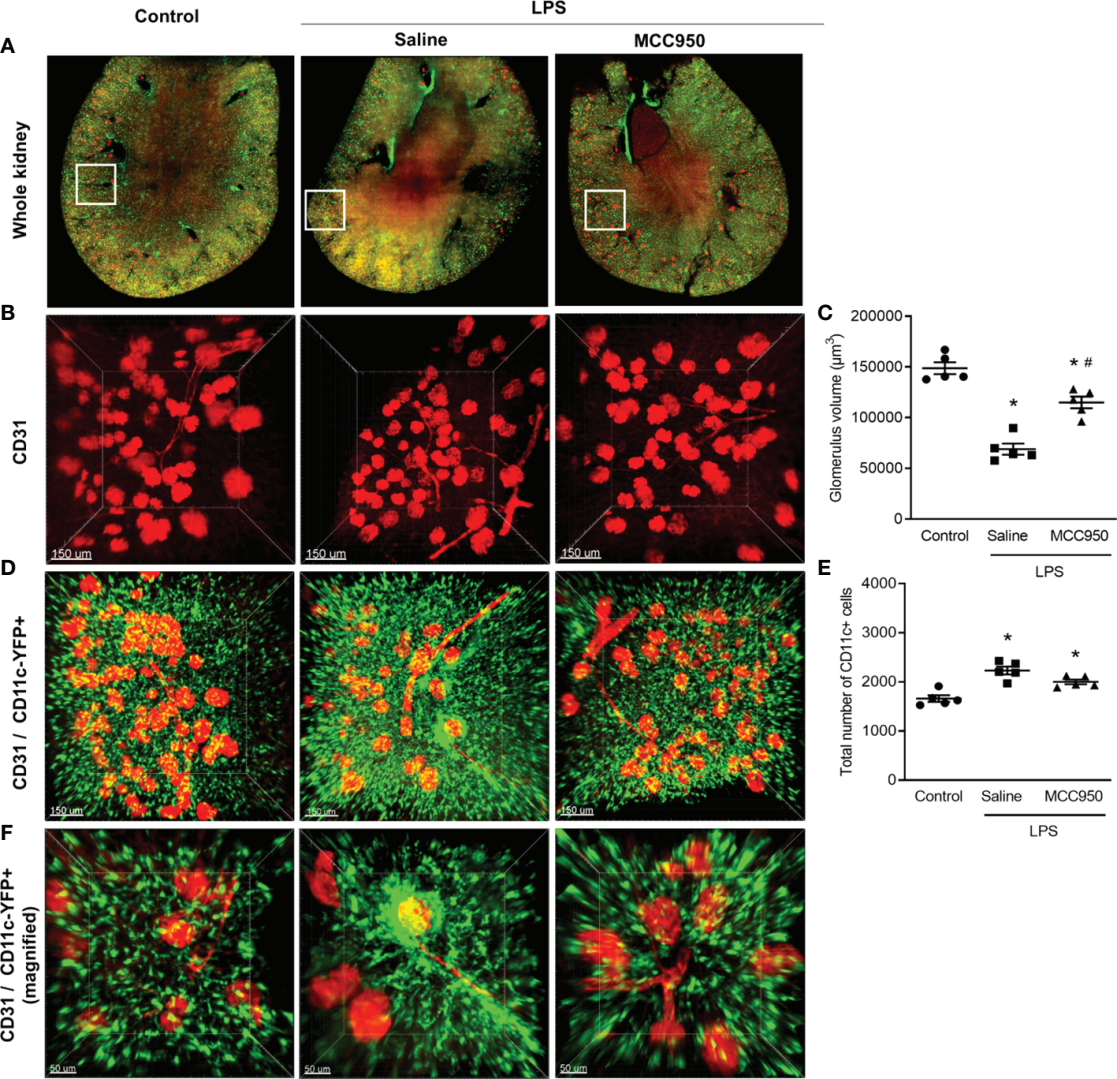
Alteration of the spatial distribution of renal mononuclear phagocytes and vasculature after LPS treatment. **(A)** LSFM image of the whole kidney. **(B)** CD31 staining. **(C)** Glomerulus volume measured from CD31 staining. **(D)** Merged image of CD11c-YFP and CD31 (scale bars, 150 μm). **(E)** Total number of CD11c-YFP+ cells. The total cell number was counted per field of view. **(F)** Merged image of CD11c-YFP and CD31 (magnified view of periglomerular and perivascular lesions, scale bars, 50 μm). Data are represented as the mean ± SEM (n=5 per group). *P<0.05 *versus* the normal control, ^#^*P*<0.05 *versus* the saline-treated LPS group.

The main strength of LSFM is rapid, wide-field image acquisition at a high spatiotemporal resolution, enabling whole-organ imaging (17). We applied LSFM to visualize the spatial distributions of CD11c+ cells and vasculatures in transversely sectioned whole mouse kidneys after clearing (**Figure 2A**, whole kidney). CD11c-YFP+ cells within a 2-mm-thick whole kidney image were mainly distributed in the cortex and, in particular, in the tubulointerstitial area. An Alexa Fluor 647-conjugated antibody to the endothelial cell adhesion molecule CD31 stained vasculature including glomeruli. Merged images of CD31-stained and CD11c-YFP+ cells were suitable for visualizing renal MNPs and glomeruli distributed in the renal cortex. In the saline-treated LPS mouse kidneys, the glomerular volume measured by CD31 staining was significantly decreased but was not affected in the MCC950-treated LPS group (**Figures 2B, C**). This finding could be related to the systemic vasoconstrictive effect after LPS administration. We compared the spatial distribution of CD11c-YFP+ cells and vasculature between the LPS-treated and control groups in 3D tissue blocks from cleared kidneys (**Figures 2D, E**). The total number and spatial density of CD11c-YFP+ cells were significantly increased in the saline-treated LPS group. Although the spatial cellular density appeared to be decreased in the MCC950-treated LPS group, there was no significant difference in the number of CD11c-YFP+ cells between the two groups. In addition, denser clusters of CD11c-YFP+ cells were found in certain sites, such as periglomerular and perivascular lesions, in the saline-treated LPS group compared to the control group or the MCC950-treated LPS group (**Figure 2F**).

### Deep and 3D Assessment by MPM

**Figure 3:**
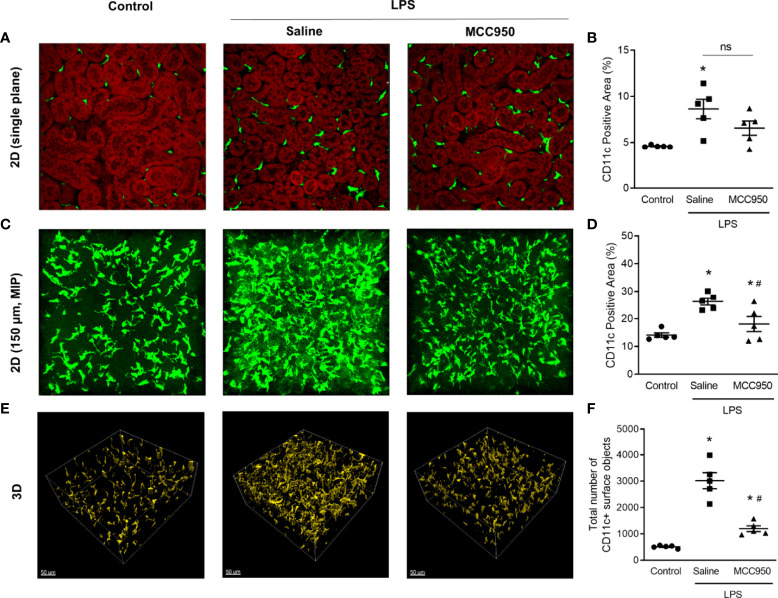
Three-dimensional visualization and quantification of CD11c+ MNPs with MPM. **(A)** Two-dimensional single plane images. The red signals indicate the autofluorescence of tubular cells. **(B)** Areas of CD11c+ cells in single plane images. **(C)** MIP images. **(D)** Areas of CD11c+ cells in MIP images. **(E)** Three-dimensional images. **(F)** The number of CD11c+ cells in 3D images. The total cell number was counted per field of view. Data are represented as the mean ± SEM (n=5 per group). *P<0.05 *versus* the normal control, ^#^*P*<0.05 *versus* the saline-treated LPS group. ns, nonsignificant; MIP, maximum intensity projection.

MPM enables deep optical imaging at a subcellular resolution with reduced phototoxicity (18). We applied MPM to analyze renal MNPs, including deep-level specimens, with a 3D approach. In a single-plane 2-dimensional (2D) image, similar to the current histological approach, there was no significant difference in the CD11c-YFP+ area between the saline-treated LPS and MCC950-treated LPS groups (**Figures 3A, B**). Through the MIP technique with the highest attenuation values on every view throughout the 150µm onto a single 2D image (**Figures 3C, D**), we found that the projection area of CD11c+ MNPs in the saline-treated LPS group was higher than that in the control and MCC950-treated LPS groups. This difference was more obvious in 3D images. In 3D images, the number of renal MNPs could be directly measured (**Figures 3E, F**). MPM imaging demonstrated that CD11c-YFP+ cells were more densely interspersed within millimeter-thick tissue of the saline-treated LPS group than the control and MCC950-treated LPS groups. These results suggested that MPM combined with kidney clearing provides direct quantitative information on renal MNPs and covers a deeper range of the kidney.

### LPS Increased the Antigen-Presenting Activity of Renal CD11c-Positive Cells

**Figure 4:**
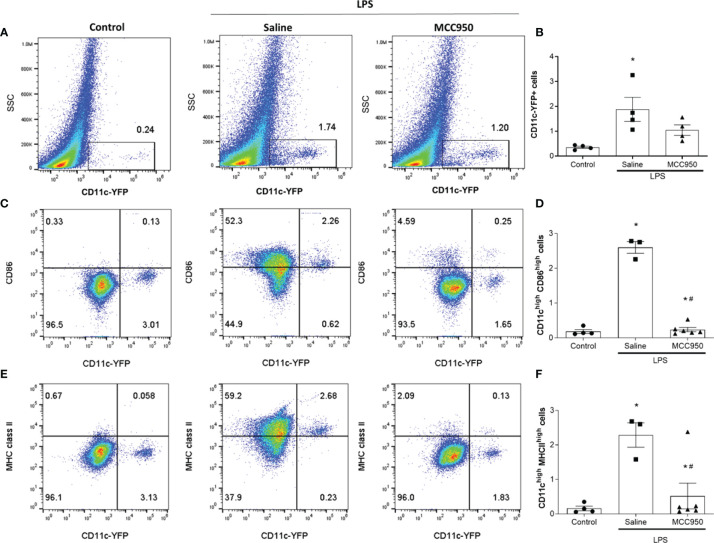
NLRP3 inhibition modulates LPS-induced surface molecule expression in renal CD11c+ cells. **(A)** Identification of CD11c-YFP+ cells by flow cytometry. **(B)** The percentage of CD11c-YFP+ cells (n=4 per group). **(C)** Identification of CD11c^high^CD86^high^ cells. **(D)** The percentage of CD11c^high^CD86^high^ cells (control group, n=4; saline-treated LPS group, n=3; MCC950-treated LPS group, n=6). **(E)** Identification of CD11c^high^MHCII^high^ cells. **(F)** The percentage of CD11c^high^MHC class II^high^ cells (control group, n=4; saline-treated LPS group, n=3; MCC950-treated LPS group, n=6). Data are represented as the mean ± SEM. *P<0.05 *versus* the normal control, ^#^*P*<0.05 *versus* the saline-treated LPS group. SSC, side scatter.

We also investigated the expression of surface molecules on CD11c+ cells with FACS analysis to confirm the functional changes in MNPs. The number of CD11c+ cells was increased in the saline-treated LPS group compared with the control group (**Figure 4A**). The number of CD11c+ cells in the MCC950-treated LPS group was not significantly reduced compared with that in the saline-treated group, as shown by FACS (**Figure 4B**). MHC class II molecules are expressed on antigen-presenting cells such as DCs, and their main function is to present processed antigens to CD4+ T lymphocytes. CD86 is a costimulatory molecule involved in the activation of naïve T cells. The proportions of CD11c^high^CD86^high^ and CD11c^high^MHCII^high^ cells were significantly increased in the saline-treated LPS group compared to the control group (0.13 vs. 2.26% and 0.06 vs. 2.68%, respectively, **Figures 4C–F**). However, MCC950 treatment significantly reduced the expression of CD86 and MHC class II molecules on CD11c+ cells (2.26 vs. 0.25% and 2.68 vs. 0.13%, respectively). FACS analysis showed significant changes in renal CD11c+ cell activity rather than the number of renal CD11c+ cells after LPS injury.

### Morphometric Analysis of Renal CD11c-Positive Cells Revealed Functional Activation After LPS Administration

**Figure 5:**
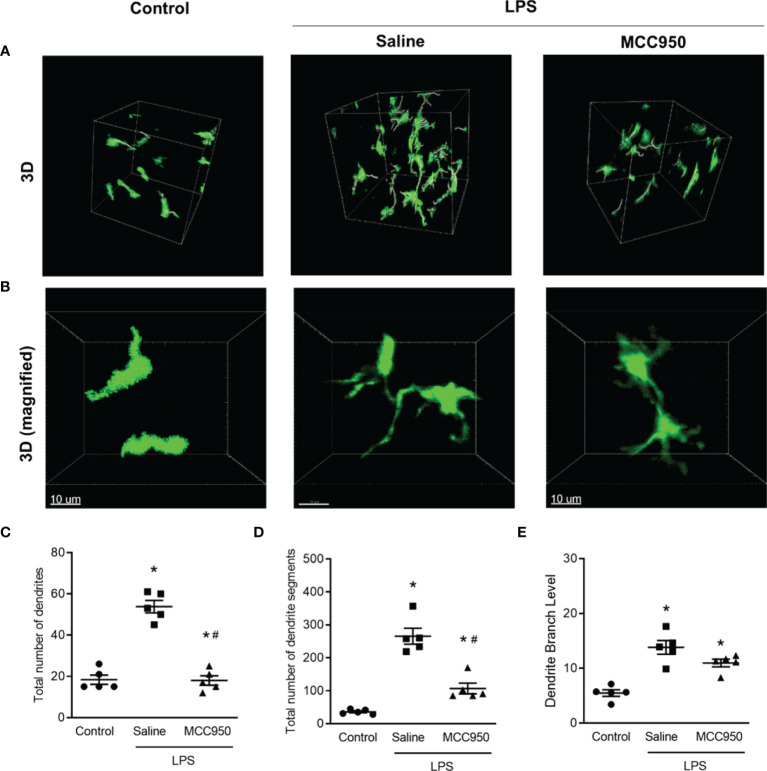
Morphometric analysis of dendrites of CD11c+ cells in the LPS-induced AKI model. **(A)** Three-dimensional MPM image. The lines in cell bodies represent the tracings for dendrite segments. **(B)** Magnified images of individual cells in the selected area. **(C)** Total number of dendrites. **(D)** Total number of dendrite segments. **(E)** Dendritic branch levels. Data are represented as the mean ± SEM (n = 5 per group). *P<0.05 *versus* the normal control, ^#^*P*<0.05 *versus* the saline-treated LPS group.

CD11c+ cells have multiple dendrites on the cell body. Dendrites have been shown to be engaged in efficient antigen uptake, antigen presentation, and interaction with T cells (19–21). We measured the number of dendrites and dendrite segments and the branch levels of CD11c+ cells to confirm that the morphological changes of MNPs reflect functional activation after LPS by using the Imaris filament tool. In 3D imaging, CD11c+ cells had multiple dendrites with branching, especially in the saline-treated LPS group compared to the control group (**Figures 5A, B**). Quantitative measurement of the dendrite and segment numbers and the number of branch levels also revealed increases in the saline-treated LPS group (**Figures 5C–E**). However, NLRP3 inhibition by MCC950 treatment prevented these morphological changes due to LPS-induced injury.

## Discussion

Here, we characterized the dynamic morphological and behavioral changes of renal resident MNPs in an LPS-induced AKI model using the renal clearing method. In this study, intrarenal CD11c+ MNPs showed an increase in cell number with more dendrites and expressed mature cell surface markers in response to LPS administration. In particular, 3D morphological assessment with LSFM and MFM after tissue clearing highlighted vital changes in the spatial distribution and morphometric measurements of individual MNPs compared to 2D assessment. Additionally, we tested the effect of MCC950, a specific NLRP3 inhibitor, on intrarenal CD11c+ MNPs and confirmed that MCC950 attenuated LPS-induced functional changes of CD11c+ MNPs involving T cell activation along with suppression of MHC class II and costimulatory molecule expression. This effect was also linked to the improvement in renal function after LPS injury.

Mononuclear phagocytes, which consist of circulating monocytes, macrophages, and DCs, are closely involved in the pathophysiology of various kidney diseases, including AKI (1, 22). Renal resident MNPs are a major component of intrarenal immune cells, which account for 46% of total CD45+ cells in normal mouse kidneys (23). Resident MNPs in the kidney act as sentinel cells in host defense against various pathogens and tissue homeostasis after renal injury (24). Our LSFM and MPM imaging showed an extensive, dense network of dendritiform cells throughout the renal cortical interstitium, consistent with other studies using fluorescent reporter mice (25, 26). CD11c+ MNPs are common cell types among resident MNPs and were previously classified as DCs. These cells are highly dendritic and induce T cell activation but also show a strong phagocytic capacity (25, 27). Approximately 70% of CD11c+MHC class II^high^ MNPs are identified as macrophages with specific surface markers (27, 28). F4/80+CD11c+ MNPs show functional phenotypes of macrophages rather than DCs (29). Indeed, renal MNPs cannot be simply defined as either macrophages or DCs and contain heterogeneous populations with overlapping surface markers and functional capacities (30).

Most previous studies on renal MNPs used single 2D sections to analyze and quantify MNP morphology and behavior. Given the complexity of kidney structures, 2D assessment of MNPs provides limited information (31). In recent years, various tissue clearing methods have emerged as promising tools to visualize the 3D structure of organs. Basically, the tissue clearing method removes lipid and pigment contents from the kidney to obtain transparency (32). This process enables volumetric imaging analysis of intact tissue at high resolution at both the subcellular level and the whole organ scale (33). Combined with fluorescence labeling, tissue clearing also contributes to a deeper understanding of microanatomy and cytoarchitecture. In the field of nephrology, tissue clearing techniques facilitated extensive morphometric analysis of thick tissue or whole kidney regarding glomerular number and volume, tubular remodeling, and podocyte depletion (34–36). We used a recent nonorganic solvent-based clearing method to acquire 3D images of MNPs, providing simple and fast protocols compared to classical stereotactic methods (37, 38). Our 3D morphometric analysis of MNPs showed changes in cell number and morphology more clearly than in the 2D analysis, providing statistically significant results and well-visualized dendritic networks of MNPs. In particular, the number and length of dendritic branches of CD11c+ cells were estimated more accurately with this technique than in methods with a single plane section. This study utilized two types of imaging modalities, MPM and LSFM. LSFM has the advantage of allowing the rapid imaging of larger samples, enabling the visualization of whole-cleared organs (33). We used LSFM to visualize the entire spatial distribution of MNPs and the microvasculature in the whole kidney. On the other hand, MPM is useful for high-resolution imaging of localized legions, including deep tissue levels (39). Since MPM can be used for uncleared tissue and intravital imaging, MPM has been used for tracing immune cell dynamics (40). The pathological processes in LPS-induced AKI were also captured by high-resolution temporal and spatial imaging of MPM (41, 42). MPM enabled subcellular level analysis of renal MNPs in our study.

Under stimulation by LPS, levels of MHC class II and the costimulatory molecule CD86 were upregulated in CD11c+ cells, indicating the immunological maturation of the cells, possibly with enhanced antigen presenting capacity. These phenotypic changes could also indicate the M1/M2 polarization. Renal MNPs are characterized into two functionally distinct cell types: proinflammatory M1 and immune-regulatory and fibrotic M2 MNPs (43). CD86 and MHCII are representative M1-like phenotype markers. M1 MNPs activated by LPS and other DAMPs are a predominant component during the initial phase of AKI, highly expressing inducible nitric oxide synthase and proinflammatory cytokines such as TNF-α, IL-1, IL-6, and IL-12 (44, 45). Our 3D morphological analysis of renal MNPs also reflected functional activity. In previous studies using intravital MPM, unilateral ureteral obstruction and IRI models generated with CD11c reporter mice showed that CD11c+ cells had a more stellate morphology with an increased number of dendrite-like protrusions (46, 47), which is consistent with our morphometric analysis. These morphological changes were accompanied by an increased capacity of MNPs to induce antigen-specific CD8 T cell activation, which is in line with the enhanced expression of costimulatory molecules in our findings. Mature resident MNPs can migrate to draining lymph nodes for antigen presentation and promote an adaptive immune response, causing greater injury to the kidney, while immature MNPs cannot (48). In our study, the number of CD11c-YFP+ cells increased in response to LPS, clustering in some periglomerular and perivascular lesions. The source of this increase in MNP number could be either resident MNPs or infiltrated monocytes. Although previous studies reported that systemic inflammatory monocytes in AKI are considered as a potential source of the renal MNP population (47, 49, 50), our study results support the pivotal role of resident renal MNPs in early AKI.

To explore the potential role of MNPs in AKI development, some studies of MNP depletion were performed with either liposome-encapsulated clodronate or transgenic mice expressing diphtheria toxin receptors. However, depletion strategies of MNP subtypes according to surface markers showed inconsistent findings (51–53), suggesting that some MNP populations are related to anti-inflammatory or renoprotective effects. Moreover, despite the pathogenic role of MNPs in AKI, whether certain therapeutic agents could affect the behavior of renal MNPs and further ameliorate kidney injury has not yet been evaluated. We evaluated the effects of the NLRP3 inhibitor MCC950 on the behavior and phenotypic maturation of MNPs. MCC950 is a specific, small-molecule inhibitor of NLRP3 that does not activate other intracellular pattern recognition receptors, such as NLRP1 and AIM2 (54). MCC950 is known to inhibit NLRP3 *via* interaction with the Walker B motif within the NLRP3 NACHT domain (11). Renal resident MNPs abundantly express all components necessary for NLRP3 inflammasome activation, producing IL-1β in response to NLRP3 agonists (6). Various DAMPs and PAMPs, including LPS, stimulate NLRP3 inflammasome assembly, thereby playing a central role in the activation of intrarenal MNPs (48, 55, 56). LPS itself can induce the NLRP3 inflammasome *via* noncanonical or alternative pathways (57). As expected, MCC950 administration improved renal function and histological findings with decreased inflammatory cytokine gene expression; given that sources of cytokine gene expression include non-immune cells, MCC950 may have also affected parenchymal cells other than MNPs (56). These findings are consistent with other studies showing that an inflammasome inhibitor attenuated intrarenal inflammation and decreased renal injury in murine models of sepsis-induced AKI, crystal nephropathy, and rhabdomyolysis-induced AKI (12, 58, 59). Moreover, LSFM and MFM analysis with renal clearing demonstrated the beneficial effects of MCC950 on the cellular behavior of CD11c+ renal MNPs in terms of cell density, spatial distribution, morphology, and phenotypic maturation. Collectively, MCC950 exhibited protective effects against the proinflammatory responses of renal MNPs in the LPS-induced AKI model. Notably, our findings provide an integrated view of the morphological, behavioral, and functional changes of MNPs in response to MCC950 in AKI, thereby linking pathophysiology and therapeutic effects.

In this study, we did not focus on the identification of a specific MNP subpopulation or the source of renal MNPs, which could be a limitation. Sophisticated classification of innate immune cells with ambiguous cell type-specific markers is not the purpose of this study. Instead, we aimed to clearly show how to apply renal clearing to evaluate renal MNPs. Further studies tracing immune cells through *in vivo* imaging will contribute to the evaluation of changing renal immune cells. Additionally, the absence of a group pretreated with MCC950 without LPS administration is a limitation. This group could provide information about the effect of MCC950 on MNPs in the healthy kidney.

In conclusion, our visualization approaches for renal MNPs using MPM or LSFM combined with kidney clearing provide spatial data, 3D quantification and intuitive insight into cell behaviors during sepsis-induced AKI.

## Data Availability Statement

The original contributions presented in the study are included in the article/supplementary material. Further inquiries can be directed to the corresponding author.

## Ethics Statement

The animal study was reviewed and approved by the Animal Research Ethics Committee of Kyung Hee University Hospital at Gangdong (approval number: KHNMC AP 2019-014).

## Author Contributions

KK wrote the manuscript and participated in the data analysis. Y-GK performed the experiment and image and histological analysis. SJ participated in the literature search. Y-GK and S-HL provided comments and interpretation of the results. S-HK performed multiphoton microscopy and 3D light sheet imaging. J-YM designed and supervised the study. All authors approved the final version of the manuscript to be published.

## Funding

This research was supported by grants from the National Research Foundation of Korea (2018R1D1A1B05049016) and a 2019 research grant from the Medical Science Research Institute of Kyung Hee University Hospital at Gangdong.

## Conflict of Interest

The authors declare that the research was conducted in the absence of any commercial or financial relationships that could be construed as a potential conflict of interest.

## Publisher’s Note

All claims expressed in this article are solely those of the authors and do not necessarily represent those of their affiliated organizations, or those of the publisher, the editors and the reviewers. Any product that may be evaluated in this article, or claim that may be made by its manufacturer, is not guaranteed or endorsed by the publisher.
